# The Murderous Coronavirus: Data and Statistics to Die or to Adapt, But Together – That is the Question

**DOI:** 10.1177/0896920520974080

**Published:** 2020-12-18

**Authors:** Pali Lehohla

**Affiliations:** University of Johannesburg, South Africa

**Keywords:** coronavirus, poverty, statistical institutions, 2020 Round of Population Censuses and information technology

## Abstract

Coronavirus is a health pandemic that threatens to spawn an economic depression. The paper explores how the official statistics constituency has been affected inter alia in its readiness of, responses to and requirements for addressing coronavirus by official statistical agencies. First, in the production of statistics and second in measuring the impact of coronavirus in society. The paper sheds light on how the virus has attacked the very lens of observation – statistics as content and statistics as an institution. In particular we explore how the pandemic disrupted the 2020 Round of Population Censuses and what country responses are. In this regard, the responses of countries would be explored from the logistical and operational readiness and adaptation. We will explore how the statistical lens has been used to understand the effects of COVID-19 on well-being. The results of Alkire–Foster method (see OPHI, 2020) that generates multidimensional poverty index will be shared.

## Introduction

Coronavirus manifested itself in an uneven world. It exposes now as we know that whilst we are existing on one globe, all is not well in this village. It threatens national commitments towards the achievement of global goals of sustainable development. Countries are faced with several questions, and inter alia, what policy options they might follow? What statistics and measurement instruments they should now use? And what a new world order should look like? These challenges seemingly are long-term but have now been summoned as immediate. The impossible has just become the inevitable. In the nine sections of the article we explore country experiences and responses to coronavirus.

## The Scourge of Coronavirus

**Figure 1.**
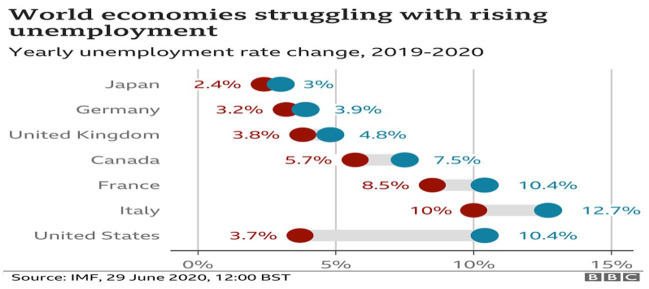
Onset of coronavirus on unemployment.

The outbreak of Coronavirus into a global pandemic drove stocks below levels last seen in 1987 according to Jones et al. (2020: **Online**). The authors further elaborate that jobs have been lost in droves as observed in Figure 1. The largest loss was in the United States where the surge in losses moved from 3.7%, the lowest level of unemployment, to 10.4% within a year.

**Figure 2.**
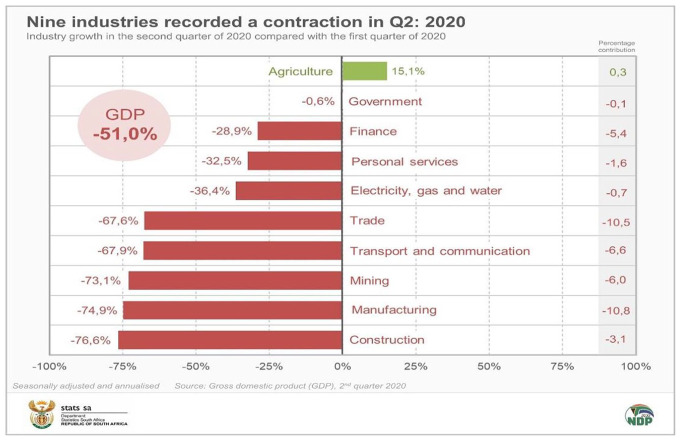
SA 2020 second quarter GDP annualized and seasonally adjusted (Statistics South Africa).

France furloughed 41% of its employed. Flights dropped from 200k per day to about 50k per day with the outbreak of Coronavirus. Oil prices dropped from a high of US$70 per barrel to as low as US$20. Consumer confidence slumped in instances by as big a margin as 80%. This was the case in Mexico. In South Africa, the second quarter seasonally adjusted annualized GDP fell steeply by 51%. This followed on a negative first-quarter GDP, which was accompanied by downgrading South Africa economy to junk status (Figure 2).

**Figure 3.**
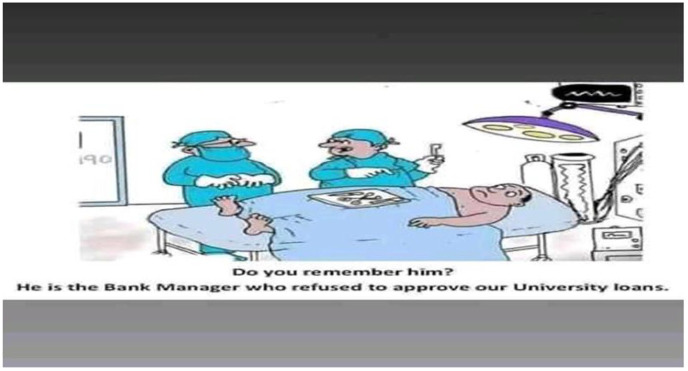
Health and education matters.^1^

Despite the supply and demand side declines in growth and consumption expenditure, Coronavirus taught the world that health matters. The health sector witnessed an astronomical rise in production and expenditure of pharmaceuticals. This included expenditure related to the race in search for vaccine. The pandemic accelerated consumption of medicines globally. However, Medical Aid Schemes are likely to experience a decline in profits because of massive and simultaneous insurance payouts. According to Businesstech (2020: Online) the Discovery Medical Aid Scheme in South Africa reported a plunge in profits of up to 94%. What comes into sharp relief is that the World Health Organization (WHO) according to Deutsche Welle (2020: Online) on June 12 urged countries and renewed its call to countries and pharmaceutical companies for the vaccine against the pandemic to be considered as a global good. Unlike the situation that prevailed when antiretrovirals for treatment of HIV and AIDS prolonged the conflicts on pricing, a vaccine for coronavirus seems to have shaped the discourse towards delivering it as a commons (Figure 3).

For instance, in the case of HIV and AIDS, Benzaken et al. (2019: Online) say that ‘Dolutegravir as a first-line drug was only made sustainable following intense negotiations between the Brazilian government and pharmaceutical industries, leading to a 70.5% drop in prices’. I-Base (2007: Online) says South Africa complained that the HIV/AIDS treatment was unaffordable at US$30 per month and advanced arguments for licensing generics. Does this then set a new tone for the role of health policies and interventions in the context of human rights?

Yet as we come to realize, The SBSNews (2020: Online) reported that according to Oxfam 51% of the vaccine has been bought on the book by 13% of the world population. The rich nations have not heeded the call.

In Africa, the pandemic was a latecomer, lagging by up to two months behind Europe and nearly four months behind China in particular and South East Asia more generally. However, South Africa became a clear outlier. Aljeezera (2020: Online) reported that daily infection rate of around 13,000 people came fifth in the World by 12 July and counting. Per capita amongst the top five, South Africa ranked number one per thousand population. It had 1 in 4400, followed by the United States with 1 in 5400. Brazil came in third at 1 in 5800 with Russia and India coming at 1 in 22,000 and 1 in 49,000, respectively. By mid-August, however, the number of infections per day had declined by above 75% to just under 4000. Despite the wide range of differences in infections and ultimate death, the economic effects of the coronavirus worldwide were commonly high and sustained in most countries. This is because of the ubiquitous effects of destruction of value chains of trade globally.

## Purchasing Power Parity Per Capita GDP and Mortality

**Figure 4.**
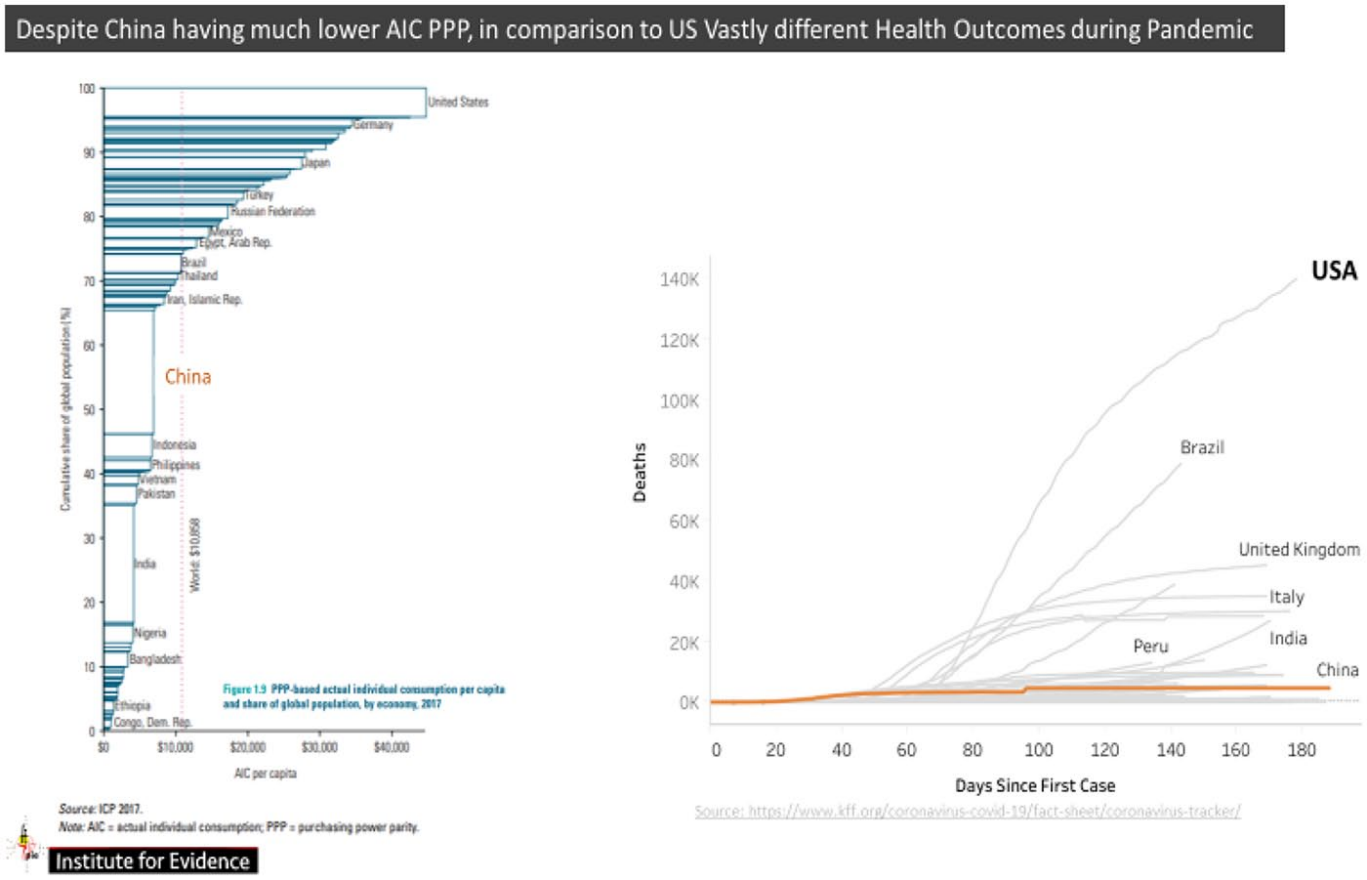
Purchasing power parity per capita and mortality by country, July 2020. Left Chart is ICP 2017 Report and Chart on the right is COVID-19 WHO.

Purchasing power parities (PPPs) are important measures of real economic performance between and amongst countries. PPPs are derived from an International Comparisons Programme (ICP) that is conducted by all national statistics offices in the world. The operation is coordinated by the World Bank on behalf of the United Nations Statistical Commission (UNSC). It is a programme that experimentally was started by universities in 1968, notably the Pennsylvania University. It is a truly global statistical operation encompassing 199 countries. Africa joined in this operation fully for the first time in 2005. The programme regionally is coordinated by regional banks and in Africa, it is coordinated by the African Development Bank. The latest results of the ICP were released in May this year and their reference date is 2017. The results cover a set of globally comparable goods and services consumed in countries by households and governments. This is particularly important in terms of calculating health expenditure per capita on a PPP basis and compare this with what the health outcomes of nations have been. Figure 4 provides insights about health expenditure by nations on a PPP basis.

Although it is pre-corona, it provides a very clear lens on the extent of vulnerability of nations as and when a pandemic strikes. Hopefully this should provide a better sense on prioritization and restore the call by WHO on the vaccine being a commons and address the warnings by van der Leyen of the European Union against ‘vaccine nationalism’.

Although coronavirus represented a global pandemic, the casualties and ultimate instance of mortality differed dramatically amongst countries. To give context of impact of COVID-19, we introduce the country PPP per capita GDP and compare these to mortality experience of countries. Figure 4 above has two components. On the left is PPP GDP per capita and on the right is the number of deaths from date of notification of coronavirus.

The United States has the highest PPP annual per capita at US$40,000 compared to China’s US$5000, yet in terms of number of deaths the United States by day 160 had recorded almost 140,000 deaths and counting against China’s under 3000 deaths. This is a figure five times the experience of China. PPPs, therefore, reflect how systems of health, education and gross fixed capital formation perform in countries. This can be compared to PPP per capita income in the United States, which is fifty times the number of deaths in China. It is clear, therefore, that how health is addressed in countries matter.

## The Advent of Information Technology

The advent and rapid developments in and of information and communication technology, especially that derived from handheld mobile sensors, remote sensing devices and transactional data from machine readable compilations, have led to major challenges, reviews and platform for changes and approaches in how national and official statistical operations are considered and undertaken. Accommodating these changes has been uneven at best and resisted at worst. These immanent developments 10 years ago prompted the United Nations Secretary General Ba Ki Moon to create what is known as the UN Global Pulse in 2009. The conception of the Pulse was in response to the global financial crisis of 2008, and the UN felt compelled to establish instruments that would generate possibilities of nowcasting. The idea was controversially considered by the United Nations Statistics Commission (UNSC) and the reception was lukewarm but largely rejected the notion. The concern of the Commission was that it did not comply with the United Nations Fundamental Principles of Official Statistics (UNFPOS). This controversial position from the UNSC notwithstanding, in 2014 the UN Secretary General again convened an initiative through a panel of independent experts to advise him on the notion of data revolution. Twenty-five experts convened and produced a report titled ‘The World that Counts’. A major outcome of the report was the creation of a facility whose forum was launched in Cape Town South Africa in January 2017 to be known as the World Data Forum. The resolutions of this fora, the very first World Data Forum, came to be known as the Cape Town Global Action Plan (CT-GAP) for Sustainable Development Data (E/CN.3/2017/3). The resolutions focused on the need for statistical organizations to adapt and transform in the face of Big Data and Data Revolution as contained in the World that Counts. This was a complete departure from the rather terse consideration to the UN Global Pulse initiative. The CT-GAP report was accompanied by a report on a series of workshops that considered a transformative agenda for official statistics (E/CN.3/2017/5). Both these documents were submitted as (E/CN.32017/35) to the 48th session of the United Statistics Commission. Their main aim was to develop instruments that would monitor the 2030 Agenda for Sustainable Development. Central to this was the need to address the deficits in the leadership role statistical systems suffered. In this regard they, the national statistical systems including multilateral ones, have to rise to the challenge and the opportunities and the potential value offered by innovative technologies in the rapidly changing environment of data sources emerging from passive and active sensors. The key question then was how would the ecosystem defined in the World that Counts be compliant to the UNFPOS. The UNFPOS were adopted as the Global Law by the United Nations General Assembly in 2014 and the view of the UNSC was that these would be compliant. What remained were the following strategic questions that would pave the way towards compliance:

1. What would the necessary transformations that the national statistical system should undertake be?
2. What would be the roadmap by which these necessary transformations to be undertaken?
3. What would be the timeframe within which these would be undertaken?
4. By what theory of change would the national statistics systems adopt to these new innovations?

**Figure 5.**
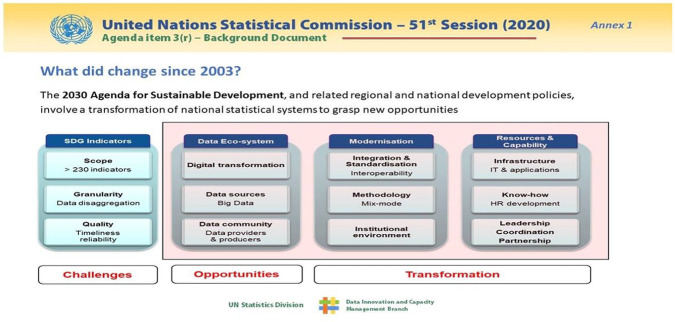
Challenges and opportunities in the statistical landscape.

As regards the transformation necessary to be undertaken by national statistics systems, an instructive energy towards this goal was put on producing a new version of a Handbook of Statistics Organisation. This will be the fourth edition from the first edition of 1954. A distinct difference is that unlike its predecessors, it will be called a Handbook on Management and Organisation of National Statistical Systems as relayed by the UNSD (2020: **Online**). In its 16 chapters it dedicates a considerable effort on 3 chapters dealing with **Data, information and knowledge management, Information Technology Management** and **Data Sources, Collection and Processing.** Under data sources, collection and processing of the following are highlighted, namely Administrative Sources, Geo-Spatial Data and Big Data. Statisticians and information technology experts had discovered the ultimate disrupter to ways of statistical collection, which would provide major opportunities for collecting information better (Figure 5).

At the 51st Session of the UNSC they prided themselves on the back of the CT-GAP resolutions that significant progress is now possible as they gear up to a transformative agenda that afforded them by opportunities the sustainable development goals (SDGs).

## The 51st Session of the United Nations Statistics Commission

**Figure 6.**
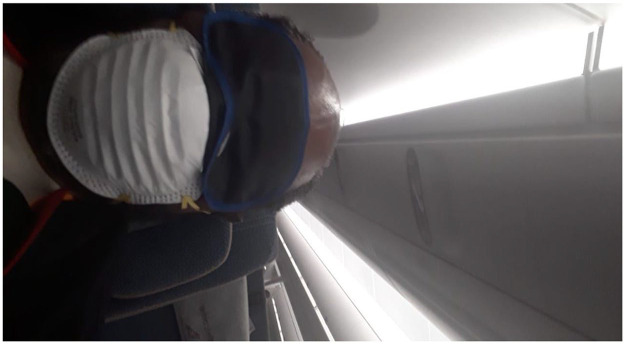
On with my mask and blind on SAA to New York, March 3.

The 51st Session of the United Nations took place from the beginning of March to the 7th of same, 2020. It occurred after the outbreak of Coronavirus in Wuhan, China. Reports were emerging that this was now spreading to Italy and Spain. Information can never be more than enough in dispelling myths and shedding light on the realities over coronavirus. At the beginning of March this year, I attended the annual ritual of national statisticians at the United Nations in New York as part of the Oxford Poverty and Human Development Initiative (OPHI) team. I bought two masks at OR Tambo and donned one immediately and throughout the flight (Figure 6).

En-route to New York I was scheduled to go to Rome where we were to focus on the Fourth UN Development Decade. There I would have presented on multidimensional poverty measures as an Oxford Research Associate and a member of the OPHI team. The session in Italy was cancelled because of coronavirus that was rearing its head in Italy.

Upon arrival at JFK Airport in New York, everyone was relaxed and there were no masks. Except for a few people there were literally no masks, although I am one not shy of feeling odd at least in my yellow suit, the mask made me feel pretty odd. So as soon as I got to my hotel, I laid it off and never to have it on again. Even on coming back to South Africa I did not know where the mask was. In New York the Chief Statisticians of China and Italy did not attend the UNSC because of the outbreak of coronavirus in their countries. The bean counters of the world seemed unconcerned about this impending danger as we exchanged pleasantries by hand shake and a hug or the Francophone Africa where the corners of the forehead bounce three times. Occasionally we would do the leg greeting to much rapturous delight. The streets of New York were very reassuring without anyone wearing a mask. We felt completely secure under the guidance of President Trump’s mighty America. There was little talk about the virus as we went on with our business, but the corridors remarked that the UNSC could be the last face-to-face meeting and the UN Women Session was unlikely to be held face-to-face. The seven days went very fast and soon it was the morning of Saturday the 7th to head home on SAA. Upon arrival South Africa was equally relaxed. On the 10th I had an appointment at the airport with the University of Zululand to discuss graduation matters and all was set to happen in May and all was reassured. The afternoon I went to present at Workplace in Sandton City, South Africa and Ashraf Garda was our host. But before we could present Ashraf asked Stafford Masie, the CEO of Workplace, to address us. We recorded what he presented at pace, anxiety all over his face as he said that within days the world will change including that the session I was presenting on was the last to be hosted there as they ready themselves to close for business. He said we have just been in a global meeting with doctors who are saying they have no clue about what is ravaging and people dying as they did. It was a sobering 10-minute rendition. And a penny then dropped, having just been to bustling New York. Yet throughout the meetings at the UNSC, we were concerned with measurement except that measuring in the context of coronavirus, which had started wreaking havoc in China and parts of Europe, was not part of the agenda. Twenty days later the world including the United States, where we had convened, started closing borders and locking down social and economic activity.

## Coronavirus the Ultimate Disruptor – 2020 Round of Censuses

**Figure 7.**
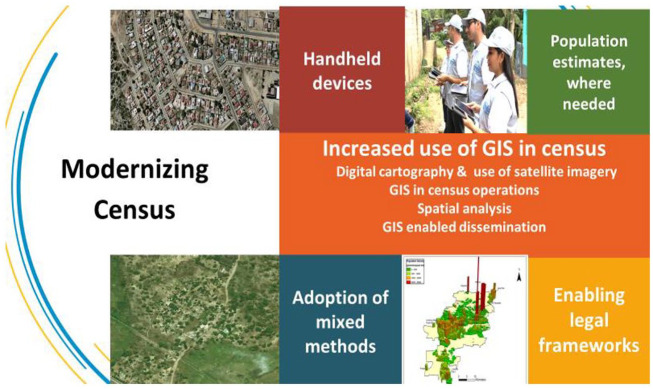
Modernization agenda for the census (UNFPA).

Statisticians and information technology experts thought and confirmed at the 51st Session that they had a lot under control, especially the technology for census taking (Figure 7).

The modernization agenda in any statistical office is usually brought through the census and massive resources are mobilized for this undertaking. These range from managing and leading the process, designing methods and technologies that will underpin the logistics, and ensuring that the attention of the public is drawn towards the census so that they can participate. It is indeed the biggest mobilization in times of peace. However, Coronavirus had a different plan. It disrupted the peacetime mobilization as it shared the space and upstaged the census. It was the ultimate disrupter. The decennial censuses for the 2020 Round of Housing and Population Censuses that began from 2015 and end in 2024 have faced their fiercest challenge since the Second World War where countries and government defaulted in keeping to the specified rhythm. However, this preceded the establishment of the United Nations and the formation of the UNSC that declared the rounds. Figure 9 is the distribution of how countries plan to undertake their census of the population by year. The modal points of undertaking a census are the year 2020 and 2021. In this period almost 130 of the more than 200 countries undertake a census. There is a lead period where of 2015–2019 a fairly sizeable number of countries undertake their censuses. Fewer at the tail-end years of 2022–2024 undertake a census.

Figure 9 shows that according to the UNFPA 44 of the countries scheduled to run a census in 2020 and 2021 have indicated that they will have to postpone their censuses. Although many parts of the world have already enlisted their populations since the year 2015 such as Egypt, Australia, Canada, Kenya, Malawi, Lesotho and Swaziland, others are yet to run the census. One of the biggest amongst them is the United States whose 24th census of the population is scheduled to have started on April 1. Other countries such as Ethiopia for a number of reasons including political disturbances have had to postpone the census at least more than once.

## Use of Technology

**Figure 8.**
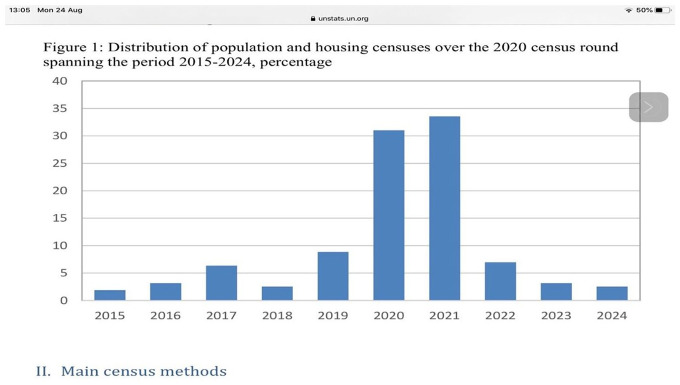
Timing of census undertaking (UNSD).

**Figure 9.**
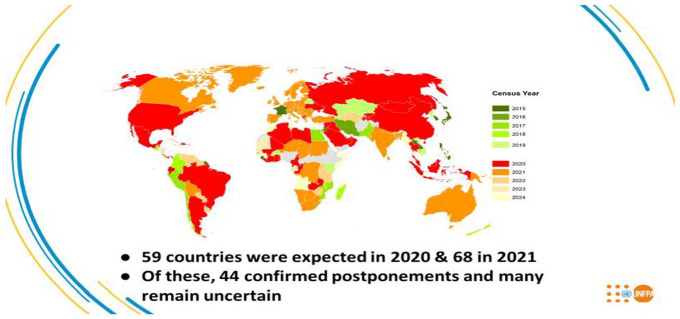
Country performance for the 2020 Round of Census.

**Figure 10.**
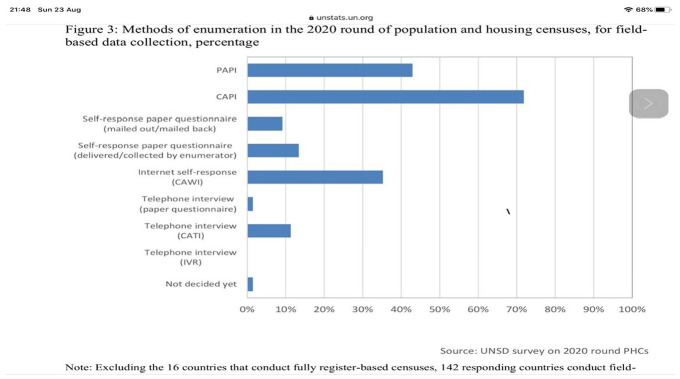
Countries by method of enumeration in the 2020 Round of Census.

A number of countries have used technology in this round compared to the 2010 round as shown in Figure 10. In Africa, Lesotho, Swaziland, Egypt and Kenya were amongst the countries that used handheld electronic devices for enumeration. In using the devices the census results that used to take two years or more to produce could be delivered within two months of completion of enumeration. This would be achieved with better geographic accuracy as well as better data quality from the respondents. As Figure 9 shows, many countries will undertake a census using modern technology that can only assist in the speed with which results are delivered; however, 2020 will pose major challenges in the execution of the census given the use of handheld devices like computer-assisted personal interviewing (CAPI). Figure 8 shows that many countries are poised to undertake a census in the year 2020 – the year of the coronavirus. As many as 310 are scheduled to run their census in 2020 and 2021, which would be during the prevalence of coronavirus. The case of the United States provides a case study of how difficult it might be to undertake a major statistical operation in the midst of a pandemic. The pandemic whilst in force makes enumeration using CAPI impractical. In developing environments, the use of paper technology could be left at the household and be picked later. However, the electronic handheld device cannot be left in the household. In the US census, they have opted for multimode collection and the precondition for this approach is adequately met. This is amongst others the availability of street addresses where paper questionnaires can be mailed and sent back to the census office. This kind of mode requires no personal contact, a condition that for many developing nations cannot be met. Should the COVID-19 persist, the developing world despite their leapfrogging of the technology wave from paper to CAPI will prove intractable. This is from a prospect of dropping and collecting the questionnaire to maximize the health requirement of keeping social distance.

## Adaptation to the Scourge of Coronavirus

We now explore case studies of the 2020 Round of Population Censuses and try to understand what lessons emerge for countries that are yet to run a census under circumstances of COVID. The United States is an important example as it brings together the health, medical, economic and political dimensions that the pandemic has raised. Other countries that will constitute the case study will be those that ran the census just before COVID-19. Kenya will be a candidate for that. The third category will be for countries that are planning a census. In this regard South Africa will be a candidate. To address measurement of multidimensional poverty in the context of COVID, we shall draw lessons from the Multidimensional Poverty Peer Network (MPPN).

**Figure 11.**
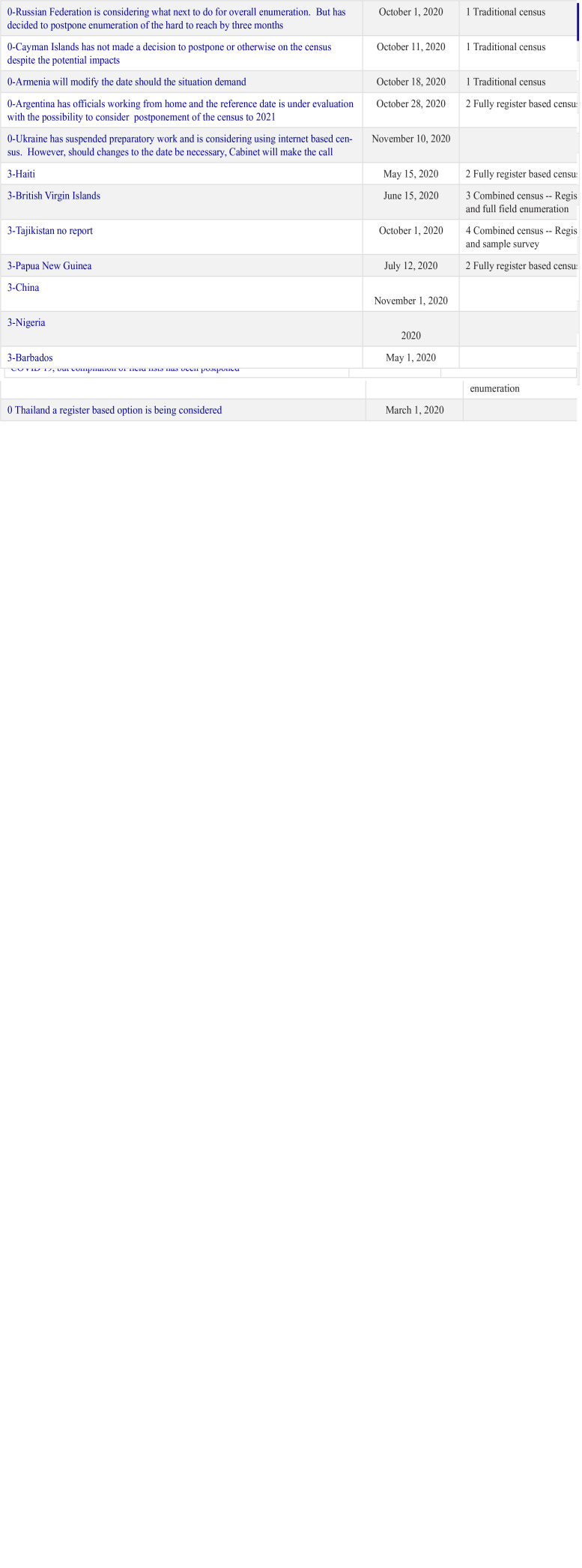
Countries by whether or not they will undertake a census in 2020 due to COVID-19.

The status of the 2020 Round of Census taking was provided by the United Nations Statistics Division and according to the report there were 57 countries that were scheduled to run a census in 2020, or the activities of 2020 got so disrupted by Coronavirus that they affected the 2021 operations adversely dispersed across the world. Each of the countries were impacted by the COVID-19. Below, we give the status of each of the countries except the United States, which we elaborated in greater detail as a case study. The countries against which the number (1) is inserted are those whose enumeration phase of the census remains unaffected by COVID-19. Those with a number (0) are those whose operations and in the main date of enumeration have been affected by COVID-19. And those labelled the number (3) are those where information at the time of compilation was not provided. Figure 11 shows that countries that have built administrative records as part of their statistical system experienced very limited impact from COVID-19. On the other hand, those that relied on traditional methods of face-to-face interaction have been severely impacted. Whilst handheld devices improved the quality and speed with which census results are released including efficient management of field operations, by virtue of the operations being face-to-face, they could not escape the disruption from coronavirus. In terms of modernization of census taking, the use of tablets and modern devices represented the most significant innovation that removed the massive logistics in managing through paper-based methods.

Let us turn to the case of the United States and cover challenges of undertaking a census in the middle of a pandemic. The United States presents almost all the phases the pandemic poses to the census. This is from health, logistics, financial, political, legal and constitutional dimensions. From that end we have taken the United States as a broad case study and elaborated it.

### The Case of the United States Census

Preparations for a census in the United States take long in the context of a Decennial Census operation. The United States has developed a sophisticated geographic base aggregated historically to the Tiger files. The census starts with identifying structures and now with remote sensing these operations have become a lot more easier. The population of the United States usually begins with the enumeration of populations in Alaska right in the middle of the winter in January. Then the rest of the country gets enlisted from April the 1st. Coronavirus will go down as one of the significant sociopolitical, technological and economic forces that troubled the United States’ landscape, particularly in the year of an election. The census questionnaire has been contested in particular in relation to the subject of citizenship. Furthermore, the President is demanding that the results get delivered per constitutional requirements, which is end of December, instead of the rescheduled period of April 2021. The scourge touched every aspect of life in the US. The United States leads the world in all aspects of the numerical expression of the pandemic. Be it in the absolute number of deaths, those infected, recoveries and/or those tested. The economic stimulus is also equally massive to deal with the effects of the pandemic. Politically, President Trump has not been outdone in his interpretation of the pandemic as a minor flu that will go away. But it was exactly around that time that the Bureau of the Census fresh from the UNSC in New York in March where the pandemic was invisible, that they as the bureau realized they could not start the enumeration, which was a mere 17 days away from April the 1st. Despite their offer for use of the internet and telephone, the tactical response to the 24th census of the United States puts paid Mandela insights into planning when he according to Lehohla (2020) Mandela said ‘Significant progress is always possible if we ourselves plan every detail and allow intervention of fate only on our own terms. Preparing a masterplan and applying it are totally different things’. The 2020 Census of the United States despite its elaborate plans has been disrupted by Coronavirus a mere 17 days to its resumption. The disruption ranged from choice of enumeration technology, personal protective equipment (PPE), logistics and medication for field staff and amplified political controversy, which usually accompany the census. The timeline of adjustments to the census operations follows below:

- 18 March 2020, Director Steven D. Dillingham suspends census field operations for two weeks until 1 April 2020 due to the COVID-19 pandemic as reported by NPR (2020).
- 27 March 2020, the agency temporarily suspends in-person interviews for its ongoing surveys, however continues to pay 2020 census employees even though field operations were supposed to be suspended.
- 28 March 2020, the Bureau further suspends field operations for an additional two weeks, to 15 April 2020.
- 27 March 2020, an employee of the Census tested positive for COVID-19, and arrangements were made to stay open with skeleton staff.
- April 13, Wilbur Ross and Steven Dillingham announce operational adjustments reactivating field operations from 1 June 2020 and extended the enumeration window for self-response to 31 October 2020.
- In addition, the change in schedule means apportionment counts will be delivered to the president by 30 April 2021, and redistricting data will be delivered to the states no later than 31 July 2021. And from then on the census operation became a PPE procurement site.
  - 4 May 2020, the Census Bureau ordered PPE for all field staff at $5,001,393.60, sanitizer worth $57,390.00 and on May 13, another contingent of sanitizers worth $557,251.20.
  - On 21 May 2020, two further procurement for $1,502,928.00 and $7,053,569.85 hand sanitizers.
  - 22 May 2020, saw two additional contracts, one was a disinfectant wipes contract for $3,137,533.00 and the other for $2,107,000.00 for blue nitrile gloves.

Coronavirus did not ease the burden of the Census Bureau, which was already embroiled in the technical matter of which questions to add in the census. In particular the question relating to citizenship was important. The citizenship question had always been included until 1950 when it was removed. But the political establishment wanted this question included in order to address the question of illegal immigrants and federal allocations. The bureau decided against it but as late as July 2020 President Trump signed a memo to the Department of Commerce, ‘Memorandum On Excluding Illegal Aliens From the Apportionment Base Following the 2020 Census’ with instructions not to include undocumented immigrants in the census totals for purposes of apportionment. So the Census of the United States does not only face the disruptive consequences of Coronavirus but also face the wrath of economic austerities that have to be implemented post the census, in particular against illegal immigrants.

The funding level for the census was determined in 2010 and firmed up in 2018. The main issue was it should not exceed the 2010 census expenditure. It was thus fixed to US$12 billion whilst the actual 2020 census budget came in at US$15 billion. With coronavirus and procurements that had to be undertaken for addressing potential coronavirus infections, it is yet to be seen how far the US$12 billion will be stretched to provide an accurate census count for the United States. The United States will continue developing an interesting perspective to census, especially in the light of the presidential election in November. President Trump has already made it clear that he needs the results of the Census per Congressional Requirements, but the director of the Bureau has already pointed out that the pandemic has disrupted programs and it is not possible to deliver the results as per legislative requirement. The last day of the count is September 30.

### The Case of South Africa

South Africa has a long then in history of running censuses prior to apartheid and continued to do so under apartheid to date. Its next Census is scheduled for October 2021. In the post-apartheid era, South Africa conducted three censuses. These were in the years 1996, 2001 and 2011. South Africa has also conducted two rounds of large-scale surveys, namely in 2007 and in 2016 to compensate for data requirements in the intervening years. The law stipulates that a census should be run every five years. In the large-scale sample survey designed to reach out to 1.5 million households, Stats SA, the legal entity that manages matters statistics in the state, used CAPI in the large-scale survey to amazing success. Within a month of completing the survey, the results were released. With the challenges of coronavirus, in preparation for the 2021, Stats SA will run a multimodal operation of internet and direct face-to-face using CAPI. To achieve maximal results from an internet-based operation, South Africa requires a robust base of physical addresses. This is a weak link for this ambition.

### The Case of India

We now turn attention to the biggest democracy on earth, India, scheduled to undertake a census in 2021, with the preparatory phases being disrupted. On the 16th of September the Indian Parliament was informed that India suspended its census preparations, which were scheduled for the 1st of April extending to September 30. This postponement was necessitated by coronavirus outbreak according to The Indian Express (2020: Online). The census of India is scheduled for 2021. The 2021 census will be the 16th census of the country. A key feature of the 15th census of 2011 was to include the enumeration by caste so that the socioeconomic determinants could be investigated and this was done for the first time since the census of 1931. The problem encountered was that the caste classification depended on self-classification and it generated too many castes and this became meaningless. For the 16th census India proposes to use the concept of other backward castes as provided by lists of castes by the states.

The importance of this Indian census is to ensure that historical inequities are understanding backwardness and poverty and disadvantage is the essence of the multidimensional measures of poverty, which are based on Amartya Sen’s notion of deprivations. Sabina Alkire and James Foster developed the Alkire–Foster method of measuring poverty multidimensionally and have through the OPHI (2020) brought the methodology to its practical uses through an MPPN of more than 70 countries. What is important is that the multidimensional poverty index (MPI) is now an official measure of indicator 1.2.2 of the SDGs, which is eradicating poverty in all its dimensions. Under the lockdown OPHI convened countries to gather information on how they have fared in terms of measuring poverty under the COVID-19 environment.

## Measuring Poverty Under COVID-19

Measuring poverty multidimensionally requires a stable data infrastructure and plays a crucial role in that it forms the base through which small area estimates of deprivation can be derived directly from the data or can through a regression model be estimated from the depth of survey data variable and the breadth of census data. Without a census the SDGs are not achievable and in particular the idea of leave no on behind is impractical. Coronavirus as we have observed threatens the ability of countries to undertake censuses except for countries that have developed registers. This threat, therefore, has direct implications on poverty measurement. We now turn to experiences that were shared through the OPHI as captured in Dimensions (2020).

In this set of case studies we discuss how different countries applied the tools to measure poverty particularly in the period of COVID-19.

### Colombia

**Figure 12.**
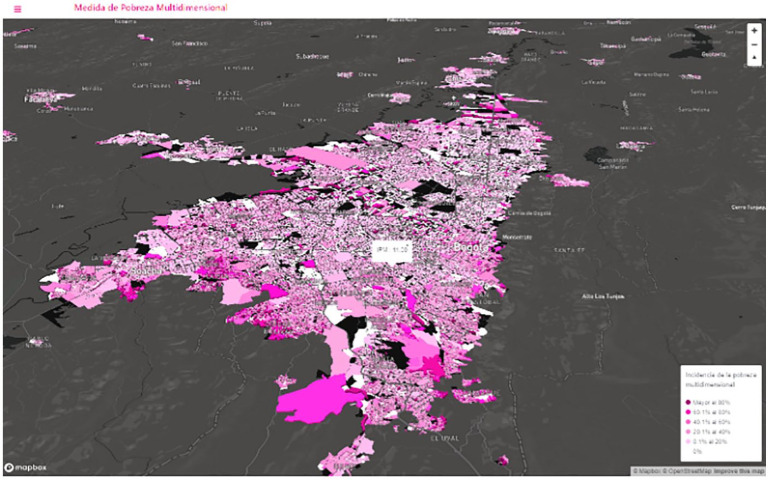
Bogota city level map of multidimensional poverty.

Oviedo (2020) and Dimensions (2020). Oviedo and Vargas of (DANE) argue that Colombia mitigated the effects of the pandemic through a collaborative effort across a number of institutions. In the main the Colombian National Statistics Office (DANE), the Ministry of Planning and the analytics team at the Institute for Technological Evaluation in Health. They jointly developed a Vulnerability Index. The base data for this was the enumeration area data recently completed in Census of 2018 (Figure 12).

They calculated this index at a neighborhood level, including at a level lower than an enumeration area such as a block of flats using demographic and comorbidity data. This method modelled the identification of the location of the population that would be at higher risk of COVID-19 complications. The model index was then mapped across space. The overlay matched other important data and most importantly the multidimensional poverty indicator generated from census data. In this regard 5 dimensions of poverty were derived from 15 poverty indicators from the census. Other facilities information was also available, such as hospitals, hotel location and daily mobility patterns during the imposed strict lockdown. The government could use these complex sources of data to design relief programmes to combat the impact of COVID-19. Such policies were the creation and deployment of solidarity income, preferential sales tax rebates to some of the poorest people in the country, and how to phase in the reactivation of the economy in cities such as Bogotá.

There could be concerns on matters privacy of citizens information, which will need to be addressed as information gets shared at that level of detail. However, what Colombia as a case study demonstrated was that during the COVID-19 crisis, data and statistics were at the centre of directing policy. But more importantly through addressing poverty a post COVID-19 period may focus on designs that should be different to the pre-COVID-19 policy outcomes and designs. It was possible to deploy the institutional and legislative framework of the Economic, Social and Ecological Emergency Decree issued in March 2020, and we have used the indicators of the MPI to target resources. **‘**Specifically, **in** those households and families that were not beneficiaries of conditional cash transfer programmes such as Families in Action or Youth in Action’, Oviedo (2020) stated.

### Chile

Alejandra Candia and Macarena Alvarado of Ministry of Social Development in Dimensions (2020) of Chile compliment the MPI as an essential tool for interrogating and surfacing poverty and vulnerability. They note that overcrowding affects almost 10% of Chlilean households, whilst about 29% have no form of any social security. The multidimensional mapping of these attributes guided government and policy makers on the challenges of social distance regulations aimed to control the spread of the virus.

### Mexico

José Nabor Cruz and Alida Gutiérrez, CONEVAL in Dimensions (2020), open up how they had to deal with the challenge of COVID-19 using the MPI. In this regard Mexico could direct social policy in understanding the magnitude of extreme poverty and by how much it could grow as a consequence of COVID-19. This information included other attributes such as access to clean water, social security and overcrowding. The information was spatially represented to understand and anticipate the evolution and progression of the pandemic given the exposure in particular of this sector of the population to abide by the prescripts of social distancing.

### Bhutan

Azusa Kubota, UNDP Bhutan in Dimensions (2020), took a look at what the economic sectoral implications would be in Bhutan. Besides Bhutan being the foremost country advancing multidimensionality as an important approach to measuring poverty and having generated the happiness index, it is important to see how tourism, the mainstay of Bhutan’s economy, was affected by COVID-19. Using a telephone survey Bhutan with OPHI mounted a Rapid Socio-Economic Impact Assessment. Through this they made a finding that over 80% of the respondents reported facing three or more deprivations simultaneously. These findings suggest severe impact, especially to employees with little or no economic security, especially savings.

### Arab Countries

Khalid Abu-Ismail and Vladimir Hlasny, UN Economic and Social Commission for Western Asia in Dimensions (2020), answer the question of lack of census data through modelling to estimate the effects of COVID-19 in the next 18–24 months. These experts are using household survey data of 2016–2018 and they simulate counterfactual probabilistic shocks of each household under each dimension within the regional multidimensional poverty measurement – education, health, housing conditions and access to services and assets. The MPI derived from this model will be projected within confidence intervals for the time limits suggested.

### African Countries

Pali Lahohla in Dimensions (2020) interacting with several African countries observes that significant effort towards finding innovative ways has been put for collecting information during the pandemic. South Africa designed a ‘long-distance’ questionnaire that gathers data on variables such as health, household income, employment and hunger – all of which contribute to poverty measurements. The Kenya census of 2019 provided a sampling frame for undertaking a panel survey to assess the impact of the pandemic on households. They generated an ID database from Census 2019 against which they matched mobile telephone subscribers. They then drew a panel and used computer-aided telephone interview to evaluate the economic impact of the pandemic on households. The survey covers indicators regarding economic activity, labour force, health, education and COVID-19 awareness.

### South Africa

Risenga Maluleke the Statistician-General of South Africa, in Dimensions (2020), says COVID-19 is clearly a huge challenge that cannot be tackled by using the same old tactics. This time is key for a healthy exchange of knowledge and learning to combine both international recommendations with local creativity. When science-based knowledge exchange is generated among countries, novel solutions can be generated; this is what countries that make up the MPPN have promoted.

The Statistician-General used the South African Multidimensional Poverty Index derived from a community survey conducted in 2016 to profile districts and municipalities. The outputs became main inputs in planning and decision-making regarding the support required in different areas of the country. In addition to that Statistics South Africa is conducting rapid online surveys to provide insights on the impact of COVID-19 with regard to health (including health behaviours and perceptions), employment, income and hunger as well as education and home-schooling, among others. The regular annual General Household Survey (GHS) that will go to field in August 2020 will carry a module to derive a COVID-19 index to inform further on the impact of COVID-19 in South Africa. Data collection for the GHS will be done telephonically.

## Conclusion

Statisticians and ICT experts thought that information technology was the ultimate game changer. Even in their 52nd UNSC session 10 days from drastic measures that the world would take against COVID-19, statisticians would consider the UN Handbook on Organisation of Statistics Office. Albeit a radical change of the title is on the offing, which will be the UN Handbook on the management and organization of a national statistics system, statisticians were conceiving of information technology as the main game changer. Little did they realize that COVID-19 would be the greatest disruption demanding major changes in the busines process models of these institutions. The disruption was so severe that it did not only become a health threat and economic threat but it rendered some of the instruments of measurement obsolete, thus demanding that those be changed. It disrupted censuses across the globe – which is the fundamental dataset for the SDGs. Countries that appear to survive are those that use administrative records. The intersection of censors and remote surveillance tools together with compilation of administrative records hold promise for statistics offices in the post corona period. Statistics and data have shown how a new world order is required and could emerge. This is particularly so in the way the countries of the world have been battered by the pandemic. Each is looking at building back better. Assessed on a PPP level it has been observed that the United States with the highest PPP per capita GDP has had the worst health outcomes from the pandemic compared to China. This put paid the notion that health is wealth and world order that will focus on wealth and education is one that will deliver sustainable development outcomes. The paper has demonstrated how the multidimensional poverty measures have become more appropriate for building back better.

## References

[bibr1-0896920520974080] Aljeezera (2020) WHO Reports Record Daily Increase in Coronavirus Cases: Live. [Online] Available at: https://www.aljazeera.com/news/2020/7/12/ [Accessed 30 September 2020].

[bibr2-0896920520974080] Benzaken S Gerson F Pereira G, et al (2019) Antiretroviral Treatment, Government Policy and Economy of HIV/AIDS in Brazil: Is it Time for HIV Cure in the Country? [Online] Available at: http://www.link.springer.com [Accessed 30 September 2020]. 10.1186/s12981-019-0234-2 PMC6694665 31412889

[bibr3-0896920520974080] Businesstech (2020) Discovery Earnings Plummet on COVID-19 Economic Fallout. [Online] Available at: http://www.bisinesstech.co.za [Accessed 28 September 2020].

[bibr4-0896920520974080] *i-Base* (2007) Agreement for Generic License of Darunavir in South Africa. [Online] Available at: https://ibase.info/htb/date/2007/05/04 [Accessed 15 September 2020].

[bibr5-0896920520974080] Dimensions (2020) Magazine on Multidimensional Poverty. [Online] Available at: https://mppn.org/dimensions/articles/ [Accessed 10 October 2020].

[bibr6-0896920520974080] Jones L Palumbo D Brown D. Coronavirus: A visual Guide to the Economic Impact. [Online] Available at: http://www.bbc.com/ [Accessed 08 September 2020].

[bibr7-0896920520974080] Lehohla P (2020) Why Do We Live with So Many Manufactured Tragedies. [Online] Available at: https://www.iol.co.za/business-report/opinion/ [Accessed 01 October 2020].

[bibr8-0896920520974080] Indian Express (2020) NPR, First Phase of Census Exercise Likely to be Delayed Due to Coronavirus: Officials. [Online] Available at: https://indianexpress.com/article/india/ [Accessed 10 October 2020].

[bibr9-0896920520974080] OPHI (2020) Medidas de Pobreza Multidimensional. [Online] Available at: https://ophi.org.uk/ [Accessed 13 October 2020].

[bibr10-0896920520974080] Oviedo J (2020) Using the MPI as a Tool for Crafting Government Responses to the Covid-19 Pandemic. [Online] https://mppn.org/mpi-tool-for-covid-19-pandemic/ [Accessed 12 October 2020].

[bibr11-0896920520974080] NPR (2020) Coronavirus Forces Bureau To Suspend Census Field Operations Until April 1. [Online] Available at: https://www.npr.org/2020/03/18/817841085/ [Accessed 11 October 2020].

[bibr12-0896920520974080] SBSNews (2020) Coronavirus: Rich Nations, Including Australia, Have Snapped Up Over Half the Future Supply of Coronavirus Vaccines. [Online] Available at: https://www.sbs.com.au/news/ [Accessed 20 September 2020].

[bibr13-0896920520974080] UNSD (2020) 51st Session of the United Nation Statistics Commission Suggested Renaming. [Online] Available at: https://www.bing.com/ [Accessed 30 September 2020].

[bibr14-0896920520974080] Wikipedia (2020) Hamlet. [Online] Available at: http://www.en.wikipedia.org [Accessed 10 September 2020].

